# Case report: a successful treatment with immune checkpoint inhibitors was associated with severe dermatologic toxicities in a patient with double primary malignancies

**DOI:** 10.1007/s12672-023-00749-5

**Published:** 2023-08-08

**Authors:** Xi Li, Yi Lei, Jiyan Liu, Hongyin Lin, Kexin Chen, Fang yin, Chunhui Wang, Hu Zhang

**Affiliations:** 1 General Practice Ward/International Medical Center Ward, General Practice Medical Center, West China Hospital, Sichuan University, Chengdu, China; 2 Department of Biotherapy, Cancer Center, West China Hospital, Sichuan University, Chengdu, China; 3 West China School of Medicine, Sichuan University Chengdu, Chengdu, China; 4 Department of Gastroenterology, West China Hospital, Sichuan University, Chengdu, China; 5 Centre for Inflammatory Bowel Disease, West China Hospital, Sichuan University, Chengdu, China; 6 Lab of Inflammatory Bowel Disease, Institution of Inflammation and Immunity, Frontiers Science Center for Disease-Related Molecular Network, West China Hospital, Sichuan University, Chengdu, China

**Keywords:** Immune checkpoint inhibitors (ICIs), Pembrolizumab, Immune-related adverse events (irAEs), Stevens-Johnson syndrome (SJS)-like eruption, Double primary malignancies

## Abstract

Dermatological toxicities are well-recognized immune-related adverse events (irAEs) secondary to immune checkpoint inhibitor (ICI) use. Corticosteroids are considered the first-line therapy for grade 3 or grade 4 skin irAEs, but long-term usage of corticosteroids may abolish the effect of ICIs. Multiple antitumor therapies might be an influencing factor in an increased incidence of skin irAEs. The safety and prognostic value in resuming ICIs after irAEs has been inconsistently reported, especially the severe skin irAE. We report a case of a 75-year-old man with non-small cell lung cancer (NSCLC) and prostate cancer with a Stevens-Johnson syndrome (SJS)-like eruption. The severe rash might have been induced by resuming pembrolizumab was successfully treated with a combination of corticosteroids, gamma globulin, and immunosuppressants. Early detection of dermatologic toxicity is crucial, especially for patients receiving multiple antitumor treatments. We should treat ICI resumption seriously after skin irAE.

## Introduction

Immune checkpoint inhibitors (ICIs) have revolutionized cancer medication by improving the outcomes of patients with various malignancies [1]. The most frequent immune-related adverse events (irAEs) caused by ICIs are cutaneous toxicities (skin irAEs), affecting 25–47% of patients and up to 72% with combination therapy [1]. The typical skin irAEs are macular rash and pruritus, while severe but rare cases are SJS and bullous pemphigoid [1]. The mechanism of immune-mediated skin toxicity is yet to be clarified, but T cells eliminate lung tumors and simultaneously attack the skin by recognizing shared antigens [1].

Pembrolizumab, the most commonly used ICI, blocks the programmed cell death-1 (PD-1) pathway in T cells and is approved for metastatic lung cancer. Few severe dermatoxic cases of pembrolizumab have been reported [2]. Herein, we report a case of pembrolizumab–induced SJS-like eruption in a patient with both primary non-small cell lung cancer (NSCLC) and prostate cancer. This case demonstrates a complete skin response to therapy with methylprednisolone combined with gamma globulin and immunosuppressant.

## Case description

**Fig. 1:**
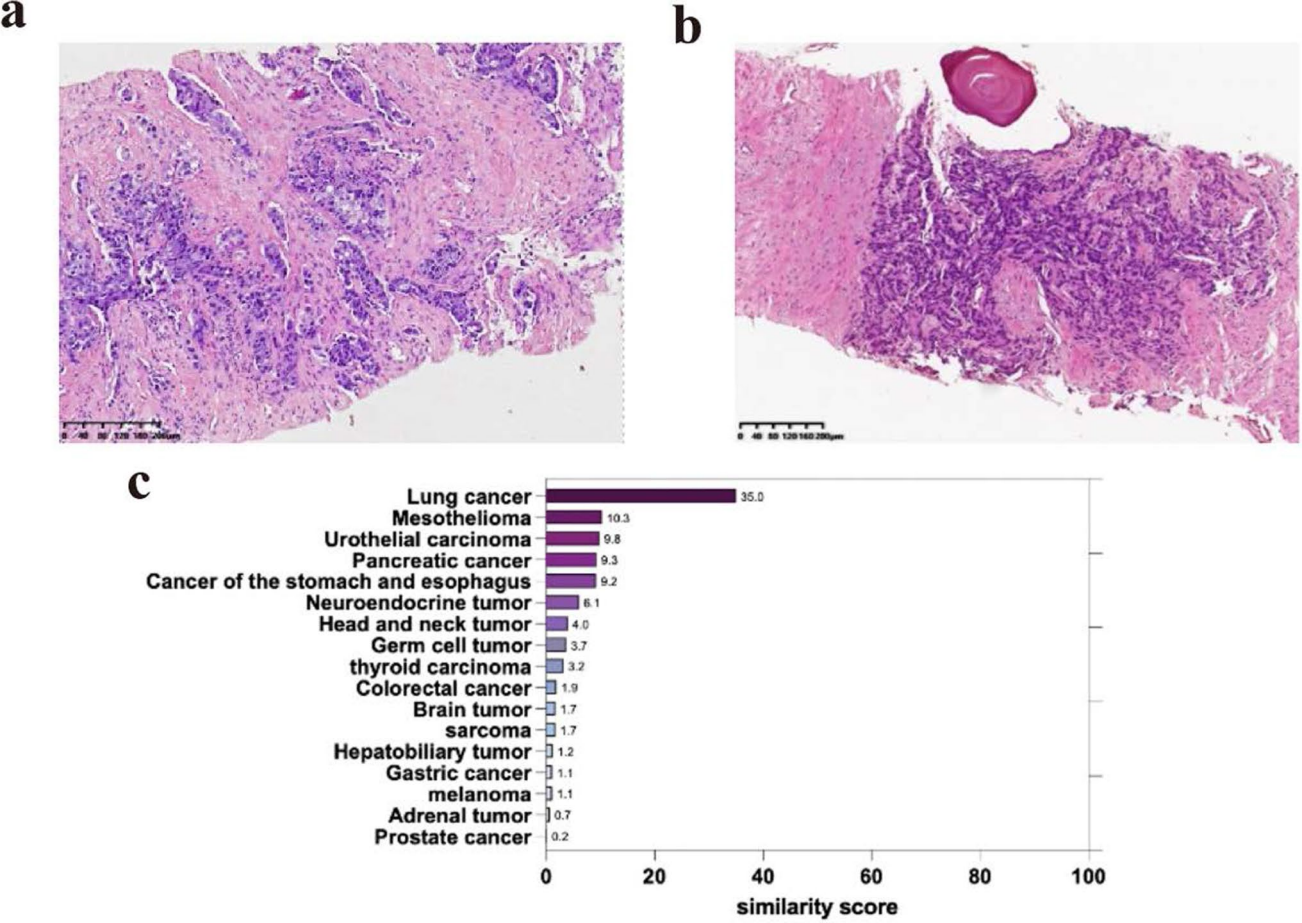
**a **Histopathological slide of the prostate: poorly differentiated prostate adenocarcinoma (HE-staining, 200 ×). Immunohistochemistry: PSA (+), AMACR (+). **b** Histopathological slide of supraclavicular lymph nodes: metastatic poorly differentiated Adenocarcinoma (HE-staining, 200×). Immunohistochemistry: PSA (–), AMACR(–), CK20 (–), CDX2(+), MLHI (+), MSH6 (+), MSH2 (+), PMS2 (+), Villin (–), TTF-1(–). **c** Detection of tumor tissue origin genes, sample: supraclavicular lymph nodes, total RNA concentration: 309ng/μl, highest similarity score:35

**Fig. 2:**
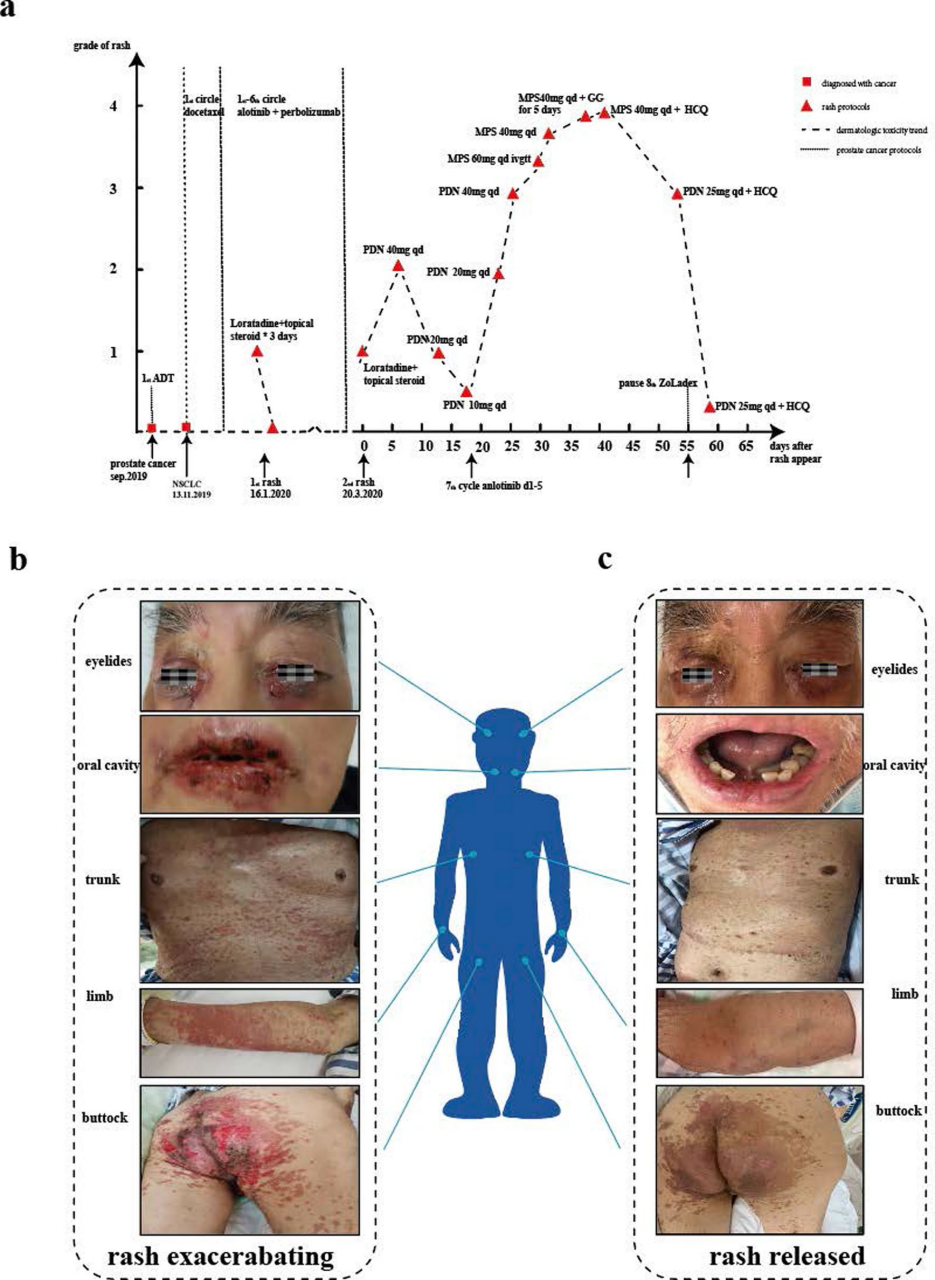
**a** Timeline of twice rash trend; the relationship between rash and antitumor regimen of prostate cancer and NSCLC. *MPS* methylprednisolone, *PDN* Prednisone, *GG* gamma globulin, *HCQ* hydroxychloroquine sulfate, *ADT* ZoLadex 3.6 mg IH every 28 days and Bicalutamide 50mg PO, day 1−day2 8. **b** Rash at different stages. A large area of red maculopapular appeared on the skin of the upper limbs, the chest, and the inner side of the left thigh. Some erythema merged into one piece, with a smooth surface, a slightly protruding skin surface, skin itching and touch pain. The rash range expanded to the head, auricle, waist, buttocks, and lower limbs. The skin of the auricle and the glans began to rupture. Large areas of erosion were seen in the lips, cheeks, and tongue. The buttocks' skin began forming a large ulceration area and peeled off. **c** The rash of the whole body gradually subsided, and broken skin and mucous membranes began to scab

A 75-year-old male presented at our hospital with dysuria following a primary diagnosis of prostate cancer (Gleason score: 4 + 4). Prostate biopsy in September 2019 indicated a highly malignant tumor (T2N0M0) (Fig. 1a). He was promptly started on androgen deprivation therapy (ADT) (ZoLadex 3.6 mg IH every 28 days and Bicalutamide 50 mg PO, Day 1–Day 28). The patient returned after one month with swollen supraclavicular lymph nodes, while the levels of both T-PSA and F-PSA/T-PSA were much better, indicating an acceptable prostate cancer treatment. The biopsy (Fig. 1b), tumor origin gene detection (Fig. 1c), and positron emission tomography (PET/CT) scan confirmed stage IV non-small cell lung cancer (NSCLC) (T3N2Mx). This patient initiated docetaxel therapy for NSCLC but experienced fatigue and severe myelosuppression. On Dec 5, 2019, the treatment was switched to anlotinib (12 mg PO, Day 1–Day 14 every 21 days) and pembrolizumab (200 mg IV every 21 days). The NSCLC was ameliorated after 2 circles of anlotinib and pembrolizumab combination treatment. However, the patient developed a grade-1 rash with multiple pruritic, erythematous lesions on the trunk and limbs. Antihistamines resolved the rash ultimately. The antitumor treatments were maintained. Figure 2a shows the treatment timeline.

After 6 circles of anlotinib and pembrolizumab and 7 cycles of ADT combo treatment, the skin rash recurred and progressively aggravated to grade-2 on Mar 20, 2020 (Day 1). The patient stopped all antitumor therapies and received oral prednisone in the hospital, significantly relieving the rash. The rash reaggravated when the prednisone dose was reduced. Despite adding methylprednisolone and gamma globulin, the rash persisted, erosions aggravated on eyelids, oral cavity, trunk, limb, and buttock and even peeled off (Fig. 2b). The circulating anti-desmoglein1, anti-desmoglein3, and anti-bullous pemphigoid 180 antibodies were negative, indicating the exclusion of Pemphigus. The patient refused a skin biopsy for personal reasons. Based on clinical presentation, laboratory test results, and medication history, an experienced dermatologist indicated that the rash was an SJS-like eruption and grade 4 skin irAEs (Fig. 2b). No other signs of irAEs were observed regarding the lungs, gut, or heart (Fig. 2b). Fearing of potential side effects, the patient refused pulse corticosteroid therapy which is the first-line treatment for severe skin irAEs. Instead, hydroxychloroquine (HCQ PO 0.2 g bid) was prescribed for the rash. Fortunately, the skin rash ameliorated completely on Day 59th (Fig. 2c).

Meanwhile, his two primary malignancies were stable by the RECIST 1.1 criteria. The patient stopped pembrolizumab and continued anlotinib and ADT. Five months later, the patient succumbed to fine-occlusive bacterial pneumonia caused by the progression of lung tumors compressing the bronchi, with no immune-related pneumonia focus.

## Discussion

Although ICIs are potent antitumor agents, they were reported in associating with various irAEs onsets, though most skin reactions are mild or moderate [3]. Unlike previous reports [1, 4] that focused on ICI-related skin toxicities in patients with a single primary tumor (e.g. lung cancer, hepatocellular carcinoma) and simple antitumor therapy, suffering from both NSCLC and prostate cancer with multiple antitumor treatments might complicate the general response of the patient and thus potentialized higher incidence and severity of skin irAEs than previously reported cases.

Whether antitumor therapies increase the risk of immune-related dermatologic toxicity is uncertain [4]. Our patient received combined NSCLC and prostatic cancer therapy, and pembrolizumab may have caused the dermatologic manifestation. Although the first rash appeared 3 months after the initial ADT, the precedented ADT-related cutaneous toxicity was not reported [5]. Moreover, the rash was not relieved after stopping ADT, hence, to blame the ADT for the rash is far-fetched. Anlotinib-related skin toxicity might cause hand-foot syndrome, affecting only the skin of limbs and its rate of causing severe rash (grade 3–4) was reported nearly 0% [6]. Double cancer and multiple antitumor therapies might be with a more complex immune milieu associated with increased specific autoimmune T-cell-mediated autoimmune skin toxic effects [7]. Consequently, we speculated our patient’s SJS-like eruption to pembrolizumab related. Skin irAEs require more vigilance as ICI usage becomes more common in combination with other cancer therapies.

The safety of restarting ICIs following an irAE remains unknown, particularly for those who experienced a severe irAE. According to the European Society for Medical Oncology (ESMO) and the American Society of Clinical Oncology (ASCO), immunotherapy can be resumed depending on the severity of irAEs, so that a rechallenge of ICIs can be considered if the rash resolves to grade ≤ 1, or if there is a tumor response or disease stability [8, 9]. Berner F et al. [7] reported a survival advantage for NSCLC patients who developed skin irAE after ICI therapy. Similarly, our patient’ NSCLC showed a positive response to pembrolizumab before irAE onset, and we resumed pembrolizumab after the first mild skin irAE resolved. However, the second skin irAE was life-threatening and required intensive care if left unattended. Unfortunately, no data exist to elucidate the relationship between prognosis and ICI resumption after skin irAEs. In contrast, Santini et al. [10] reported that 52.0% of patients who resumed ICIs experienced irAEs again, most recurrent irAEs were mild, and only a few patients with skin irAEs were rechallenged with ICIs among the 18 studies included in their work. This study further reported on the patients who resumed ICIs after irAEs. 8% had recurrent irAEs, and 21% had a new irAE, including a grade-5 SJS case. Several factors need to be weighed in deciding whether the rechallenge ICI is applicable: the tumor grading, the clinical presentation and biological status, the availability of alternative treatments, and the likelihood and severity of recurrent irAEs [11]. Future studies should investigate the association between skin irAEs and different ICIs.

## Conclusion

We report a patient with primary NSCLC and primary prostate cancer who suffered SJS-like eruptions after pembrolizumab therapy. Early detection of dermatologic toxicity is crucial, especially for patients receiving multiple antitumor treatments. We should treat ICIs resumption after skin irAE seriously.

## Data Availability

The original contributions presented in the study are included in the article/supplementary material, further inquiries can be directed to the corresponding author.
